# Eco-physiological response and genotoxicity induced by crude petroleum oil in the potential phytoremediator *Vinca rosea* L

**DOI:** 10.1186/s43141-022-00412-6

**Published:** 2022-09-20

**Authors:** Zahra S. Hussein, Ahmad K. Hegazy, Nermen H. Mohamed, Mohamed A. El-Desouky, Shafik D. Ibrahim, Gehan Safwat

**Affiliations:** 1 Faculty of Biotechnology, October University for Modern Sciences and Arts (MSA), 6th of October, 12451 Egypt; 2 Botany and Microbiology Department, Faculty of Science, Cairo University, Giza, 12613 Egypt; 3 Egyptian Petroleum Research Institute, Nasr City, Cairo, 11727 Egypt; 4 Chemistry Department, Biochemistry Division, Faculty of Science, Cairo University, Giza, 12613 Egypt; 5 Agricultural Genetic Engineering Research Institute (AGERI), Agricultural Research Center, Giza, Egypt

**Keywords:** Antioxidants, FRAP, Phenolics and flavonoids, Photosynthetic pigments, Plant growth, SCoT and ISSR markers, Tannins

## Abstract

**Background:**

Phytoremediation is determined as an emerging green technology suitable for the safe remediation and restoration of polluted terrestrial and aquatic environments. In this study, the assessment of an ornamental plant, *Vinca rosea L*., as a phytoremediator of crude oil in polluted soils was conducted. In an open greenhouse experiment, plants were raised in sandy-clayey soils treated with 1, 3, 5, and 7% oil by weight. The experiment was conducted over 5 months.

**Results:**

Total petroleum hydrocarbon (TPH) degradation percentage by *V. rosea* after a 5-month growth period ranged from 86.83 ± 0.44% to 59.05% ± 0.45% in soil treated with 1 and 7%, respectively. Plants raised in polluted soils demonstrated a dramatic reduction in germination rates, in addition to growth inhibition outcomes shown from decreased plant height. An increase in branching was observed with an increase in oil pollution percentages. Moreover, the phytomass allocated to the leaves was higher, while the phytomass witnessed lower values for fine roots, flowering and fruiting when compared to the controls. Apart from the apparent morphological changes, there was a decrease in chlorophyll a/b ratio, which was inversely proportional to the oil pollution level. The contents of carotenoids, tannins, phenolics, flavonoids, and antioxidant capacity were elevated directly with an increase in oil pollution level. The start codon-targeted (SCoT) polymorphisms and inter-simple sequence repeat (ISSR) primers showed the molecular variations between the control and plants raised in polluted soils. The genetic similarity and genomic DNA stability were negatively affected by increased levels of crude oil pollution.

**Conclusions:**

The ability of *V. rosea* to degrade TPH and balance the increased or decreased plant functional traits at the macro and micro levels of plant structure in response to crude oil pollution supports the use of the species for phytoremediation of crude oil-polluted sites. The genotoxic effects of crude oil on *V. rosea* still require further investigation. Further studies are required to demonstrate the mechanism of phenolic, flavonoid, and antioxidant compounds in the protection of plants against crude oil pollution stress. Testing different molecular markers and studying the differentially expressed genes will help understand the behavior of genetic polymorphism and stress-resistant genes in response to crude oil pollution.

**Supplementary Information:**

The online version contains supplementary material available at 10.1186/s43141-022-00412-6.

## Background

Continued human and industrial activities have caused a catastrophic accumulation of toxic contaminants in the environment. One of the most harmful and widespread organic contaminants is crude petroleum oil [1, 2]. The exploration, transportation, and processing of petroleum oil have become major sources of serious contamination problems and ecological risk for many regions and countries [3, 4]. The constituents of crude oil are mainly divided into four components: resins, asphaltenes, aromatic hydrocarbons, and saturated hydrocarbons [5]. The accumulation of these hydrocarbons may be a limiting factor for microbial proliferation and plant growth due to their hydrophobic nature, which causes a reduction in the available nutrients [6]; specifically, many polycyclic aromatic hydrocarbons are considered to have adverse effects on microorganisms and higher organisms because of their highly toxic, carcinogenic, and mutagenic properties [7, 8].

There are three common methods used for controlling petroleum hydrocarbon contamination: chemical, physical, and biological remediation. Phytoremediation is one of the biological remediation methods that has become of increasing interest owing to its distinctive advantages as a green, safe technology with low cost and wide variety of potential applications [9]. Phytoremediation is the use of the special ability of certain plants to eliminate or reduce the contamination of soils, sediments, surface, and ground water [10]. In this process, plants utilize different mechanisms in order to remediate pollutants, including absorption, transformation, evaporation, stabilization, and rhizosphere degradation [11]. Many plants have already been studied for their ability to remediate environmental contaminants, while others have shown potential as phytoremediators of heavy metals, radionuclides, and petroleum hydrocarbons [8, 12].

Start codon-targeted (SCoT) polymorphisms and inter-simple sequence repeats (ISSR) have been reported to be more accurate and reliable than other molecular markers [13]. The SCoT marker is able to detect the coding sequence polymorphisms since the designed primers anneal to the conserved region surrounding the translation start codon ATG [14]. Thus, the amplified fragments from SCoT markers may be correlated with the functional traits and their genes. Meanwhile, the ISSR markers detect polymorphisms in microsatellites, which are not necessarily functional genes [15].

Although there has been significant progress into research on petroleum hydrocarbon phytoremediation [16], the screening and characterization of crude oil phytoremediators are still ongoing and are considered a promising field of research. Using ornamental plants for phytoremediation has an advantage over using other reported types of plants because they can effectively reduce contamination and do not enter the food chain. In urban regions, ornamental plants that can successfully remediate contaminated soil can also decorate and beautify the environment [17]. The study species, *Vinca rosea*, is an important flowering and evergreen ornamental plant that has become naturalized in some coastal tropical and subtropical regions. As an economically important species, it has been used for medicinal purposes since the Middle Ages [18]. The species has been studied for its role as a phytoremediator of toxic metals and diesel exhaust [19, 20], but its role as a phytoremediator of crude oil pollution is still under investigation [20]. To support this direction and owing to its various advantages as an ornamental plant that can grow in a wide range of conditions, *Vinca rosea* L. is assessed for its potential suitability in the remediation of crude petroleum oil pollution (hereafter, oil pollution). The eco-physiological and biochemical responses of the species to oil pollutants are investigated, and the genotoxicity is assessed through the analysis of SCoT and ISSR molecular markers.

## Methods

### Experimental design

The *V. rosea* plants were raised in an open greenhouse that belongs to Cairo University for 5 months (from January to June 2020). The soil was a mixture of sandy and black soil. The chemical and physical analysis of soil demonstrating its properties is shown in Table 1. The soil analysis followed Allen et al.’s analysis method [21]. The crude oil was added to the air-dried soil in plastic pots (22 cm in diameter and 25 cm in depth) to develop oil per soil percentages of 1, 3, 5, and 7%. Five replications were used per each treatment with negative control (plants in soil without oil treatment) and positive control (soil with oil treatment only). A pilot sighting experiment was carried out at a small scale to indicate the experimental design of this study. It indicated that successful phytoremediation occurs under low levels of pollution up to 7%, whereas under higher levels *V. rosea* failed to establish healthy plant individuals. Moreover, treatments at level 100% showed zero percentage survival [8, 20, 22]. Many previous studies have shown that phytoremediation is only effective at low levels of pollution, around 5% [20]. The plant height, shoot diameter, and number of branches per specimen were recorded after 1 month of establishment and at the termination of the experimental period. Plants were watered regularly with fresh water from the Nile River. Care was taken during irrigation to avoid water leakage from the pots and soil desiccation.

**Table 1:** Soil physical and chemical properties

Soil character	Value
Gravels (wt. %)	0.85±0.13
Coarse sand (wt. %)	28.05±2.18
Fine sand (wt. %)	30.17±2.08
Silt (wt. %)	23.15±1.17
Clay (wt. %)	17.78±2.05
Organic matter (wt. %)	1.33±0.78
pH	6.13±0.15
Electric conductivity (Sm m^−1^)	24.05±1.82

### Percentage of TPH degradation

Soil samples were dried at room temperature in the dark and sieved through 100 mesh. Samples were gently homogenized prior to TPH extraction. According to Peng et al. [23], TPH were extracted from 10 g of soil in a centrifuge tube using 50 ml of dichloromethane by ultrasonication for 30 min. After centrifugation at 3000 rpm for 10 min, the supernatant was collected. The extraction cycle was repeated three times, and the supernatant was combined in a clean, dry, and previously weighed flask. In a fume hood, the flask was allowed for dichloromethane evaporation until it reached a constant weight. The TPH extracted amount was estimated as the difference between the weight of the dried flask containing the extracted hydrocarbons and the weight of the empty pre-weighed flask. This extraction method resulted in a 4.8% petroleum hydrocarbon loss. The TPH degradation percentage was determined gravimetrically [24] and calculated according to the following:

$$\mathrm{TPH}\ \mathrm{degradation}\ \left(\%\right)=(({T}_I-{T}_F)/{T}_I)\times 100$$

where TPH is the total petroleum hydrocarbons (%), *T*_I_ is the initial concentration of petroleum hydrocarbons used in the treatment, and *T*_F_ is the final concentration of petroleum hydrocarbons in the soil at the end of the experiment.

### Seed germination

Seed germination was tested in the potted soils treated with different crude oil pollution levels of 0, 1, 3, 5, and 7%. Five replication pots were used and 25 seeds per pot were sown at a 1-cm depth. A total of 125 seeds were used in every treatment level. The pots were watered every 2 days to keep soil moisture for 2 weeks. The germination data was taken based on the emergence of seedling plumules from the soil surface.

### Phytomass allocation

After the termination of the experiment, the plants were in a flowering/fruiting growth stage and were harvested for estimation of the phytomass allocation to different plant organs. Five individual plants per crude oil pollution treatment were harvested and separated into roots (main and fine roots), stems, leaves, and reproductive organs including the flowers and fruits. The material was oven-dried for 48 h at 80 °C. The dry phytomass of each fraction was weighed and calculated as the percentage of total plant weight. Five replicates were taken at random from each treatment. The root/shoot ratio was calculated by dividing the root by the shoot dry phytomass [25].

### Determination of photosynthetic pigments

Hiscox and Israelstam [26] method was followed to measure the content of the photosynthetic pigments where samples of 2 g of *V. rosea* fresh tissue were mixed with 25 ml of concentrated dimethyl sulphoxide (DMSO) and left in the dark at 4 °C for 24 h. The tissue was thawed and macerated, followed by filtration of the pigment extract by a sintered glass filter in dim light. The final volume of the extract filtrate was brought to a known volume, and the optical density was measured at 436, 440, 474, 644, and 662 nm by a UV-visible spectrophotometer (Shimadzu UV−1208 model; Canby, OR, USA). Pure DMSO was used as a blank. The data is an average of 5 replicates per sample, with values expressed as mean±SE. The contents of chlorophylls (a, b, and total) and carotenoids were expressed as μg/g dry weight and calculated according to the following equations:

$${\displaystyle \begin{array}{c}\mathrm{Chl}\ \mathrm{a}=\left[0.0127\ \mathrm{A}622+0.02269\ \mathrm{A}644\right]\times \mathrm{Dilution}\ \mathrm{factor}\ \left(\upmu \mathrm{g}/\mathrm{g}\right)\\ {}\mathrm{Chl}\ \mathrm{b}=\left[0.0229\ \mathrm{A}622+0.00468\ \mathrm{A}644\right]\times \mathrm{Dilution}\ \mathrm{factor}\ \left(\upmu \mathrm{g}/\mathrm{g}\right)\\ {}\mathrm{Total}\ \mathrm{Chl}=\left[0.0202\ \mathrm{A}622+0.00802\ \mathrm{A}644\right]\times \mathrm{Dilution}\ \mathrm{factor}\ \left(\upmu \mathrm{g}/\mathrm{g}\right)\\ {}\mathrm{Cx}+\mathrm{c}=\left[\left(1000\mathrm{A}474-1.29\ \mathrm{Chl}\ \mathrm{a}-53.77\ \mathrm{Chl}\ \mathrm{b}\right)/220\right]\times \mathrm{Dilution}\ \mathrm{factor}\ \left(\upmu \mathrm{g}/\mathrm{g}\right)\end{array}}$$

where Chl a = chlorophyll a, Chl b = chlorophyll b, Chl a + Chl b = total chlorophyll, Cx + c = carotenoids, and Ax = absorbance at x nm.

### Quantitative estimation of tannins

The collected plant material was cut into small pieces and transformed into the laboratory for air-drying, then in an oven at 50 °C for 24 h to attain constant weight. The dry plant materials were ground into powder in a plant grinder. Tannins were estimated in the plant powder by titration of the plant extract against potassium permanganate solution following the method of AOAC [27]. The aliquot of plant extract was mixed with 12.5 ml of indigo carmine solution and distilled water; the mixture was titrated versus KMnO_4_ solution to a faint pink color endpoint (*Y* ml). The volume of KMnO_4_ represents the total tannins plus all other related compounds. For the determination of KMnO_4_ volume (*X* ml) used for titration of non-tannin related compounds, another aliquot of the extract was mixed with gelatin solution soaked for 1 h in saturated NaCl. The mixture was then warmed until the gelatin had dissolved, and after cooling, the solution was made up to a known volume with saturated acidic NaCl solution and powdered kaolin [28]. The mixture was shaken for 15 min before filtration. The known volume of the filtrate was mixed with indigo carmine solution and distilled water. This mixture was again titrated against KMnO_4_ solution until a faint pink endpoint was reached. The volume of KMnO_4_ used to titrate true tannin was calculated by the values of *Y* and *X*, where tannin concentration was estimated as the following:

$$1\ \mathrm{ml}\ \mathrm{of}\ \mathrm{standard}\ {\mathrm{KMnO}}_4\ \mathrm{solution}=0.595\ \mathrm{ml}\ \mathrm{of}\ 0.1\mathrm{N}\ \mathrm{Oxalic}\ \mathrm{acid}$$

$$1\ \mathrm{ml}\ \mathrm{of}\ 0.1\ \mathrm{N}\ \mathrm{Oxalic}\ \mathrm{acid}=0.0042\ \mathrm{g}\ \mathrm{of}\ \mathrm{tannin}$$

### Antioxidants

#### Sample preparation

Plant leaves from treated and control *V. rosea* plants were oven-dried at 60 °C to a constant weight. The dry material was ground into a fine powder. Two grams of the dry plant material was mixed with 200 ml of ethanol. The mixture was soaked overnight, then samples were filtered and the collected extracts were exposed to evaporate the solvent. The Known weight of the dark green residue was weighed and dissolved in 5 ml of methanol to reach a 2 mg/ml concentration.

#### Total phenolic and flavonoid content

Gallic acid standards were prepared for the measurement of total phenolics, and rutin standards were prepared for total flavonoids. A total of 9 serial dilutions were prepared with concentrations of 1000, 800, 500, 400, 200, 100, 50, 25, and 12.5 μg/ml to prepare the gallic acid standards from the 1 mg/ml gallic acid in methanol stock solution. Rutin standards were prepared from the rutin stock solution of 1 mg/ml in methanol. A total of 10 serial dilutions were prepared with concentrations of 1000, 800, 500, 400, 200, 100, 50, 25, 12.5, and 6.25 μg/ml. The results of the samples with a concentration of 2 mg/ml and standards were recorded using the microplate reader FluoStar Omega [29, 30]. Three replications per sample were taken, and values were expressed as mean±SE.

#### Antioxidant determination by FRAP

To prepare the Trolox standard for the ferric reducing ability of plasma (FRAP) assay, a Trolox stock solution of 1 mM in methanol was prepared, and 10 serial dilutions were prepared in the concentrations of 1000, 800, 600, 400, 200, 100, and 50 μM. Samples of treated and control *V. rosea* plants were prepared at a concentration of 2 mg/ml in methanol.

The FRAP assay was carried out according to Benzie and Strain’s [31] method. The TPTZ (2,4,6-Tripyridyl-s-triazine) reagent was freshly prepared as follows (300 mM acetate buffer (pH = 3.6), 10 mM TPTZ in 40 mM HCl, and 20 mM FeCl_3_, in a ratio of 10:1:1 v/v/v, respectively). From the freshly prepared reagent, 190 μl was used and mixed with 10 μl of the sample in a 96-well plate (*n* = 3). The mixture was incubated in the dark for 30 min at room temperature. The resulting blue color was measured at 593 nm, and the results were recorded by microplate reader FluoStar Omega. Three replicate measurements were taken per sample, and values were expressed as mean±SE.

### Extraction of genomic DNA and evaluation of genotoxicity

#### DNA extraction

The fresh tissues of *V. rosea* plants raised under different crude oil treatment levels (1%, 3%, 5%, and 7%), and plants raised in clean soil (control) were frozen in liquid nitrogen and ground to a fine material using a prechilled mortar and pestle. The ground material was used for genomic DNA extraction with the DNAeasy Plant Mini Kit (Qiagen, Santa Clarita, CA). The obtained DNA was run on a 1% agarose gel and measured using a NanoDrop-2000 Spectrophotometer (Thermo Scientific, Germany) for the determination of purity and concentration. The DNA molecular marker (100 bp DNA ladder) was also loaded.

#### SCoT and ISSR-PCR analysis

To amplify the obtained DNA and detect polymorphism in treated and untreated plants, a set of 10 primers for each of the SCoT and ISSR analyses were used (Table 2). The PCR amplification reaction was carried out in a 25-μl reaction volume containing 12.5 μl (2X) Master Mix “Thermo Scientific™” 3 μl genomic DNA (10 ng), 2.5 μl primer (10 pmol), and 7 μl nuclease-free water. The PCR amplification of SCoT and ISSR cycling parameters was carried out as shown in Table 3. The PCR products were run on a 1.5% agarose gel containing ethidium bromide (0.5 μg/ml) in TBE buffer for 30 min, at 95 V for separation. A molecular marker of 100 bp was also loaded to estimate the size of the bands in SCoT and ISSR profiles. The bands were visualized on a UV transilluminator and photographed by a gel documentation system (BIO-RAD 2000).

**Table 2:** List of primers used in SCoT-PCR and ISSR-PCR

SCoT primers		ISSR primers	
Primer name	Sequence (5́′– 3́′)	Primer name	Sequence (5́′›–3́′)
**SCoT-1**	5′-ACGACATGGCGACCACGC-3′	**ISSR-3**	5′-ACACACACACACACACYT-3′
**SCoT-2**	5′-ACCATGGCTACCACCGGC-3′	**ISSR-4**	5′-ACACACACACACACACYG-3′
**SCoT-3**	5′-ACGACATGGCGACCCACA-3′	**ISSR -5**	5′-GTGTGTGTGTGTGTGTYG-3′
**SCoT-4**	5′-ACCATGGCTACCACCGCA-3′	**ISSR -8**	5′-AGACAGACAGACAGACGC-3′
**SCoT-5**	5′-CAATGGCTACCACTAGCG-3′	**ISSR -9**	5′-GATAGATAGATAGATAGC-3′
**SCoT-6**	5′-CAATGGCTACCACTACAG-3′	**ISSR -13**	5′-AGAGAGAGAGAGAGAGYT-3′
**SCoT-7**	5′-ACAATGGCTACCACTGAC-3′	**ISSR -14**	5′-CTCCTCCTCCTCCTCTT-3′
**SCoT-9**	5′-ACAATGGCTACCACTGCC-3′	**ISSR -19**	5′-HVHTCCTCCTCCTCCTCC-3′
**SCoT-10**	5′-ACAATGGCTACCACCAGC-3′	**ISSR -20**	5′-HVHTGTGTGTGTGTGTGT-3′
**SCoT-11**	5′-ACAATGGCTACCACTACC-3′	**R -9**	5′- ACACACACACACACACG -3′

*A* Adenine, *T* Thymine, *G* Guanine, *C* Cytosine, *Y* (C or T), R (A or G), *H* (A or C or T**)**

**Table 3:** The PCR reaction parameters of ISSR and SCoT

Step	Temperature		Time period		Cycles
	SCoT	ISSR	SCoT	ISSR	
**Initial denaturation**	94°C	94°C	5 min	5 min	1
**Denaturation**	94°C	94°C	40 s	40 s	
**Annealing**	50°C	45°C	1 min	1 min	40
**Extension**	72°C	72°C	1.5 min	1.5 min	
**Final extension**	72°C	72°C	7 min	7 min	1

The produced bands from SCoT and ISSR marker amplifications were scored and compared to determine the relatedness between the genetic material of the treated samples and the control. The distinct clear bands were scored for presence as (1) and absence as (0). Bands with the same mobility were considered and scored as identical. The binary statistic matrix was constructed. Dice’s similarity matrix coefficients were then calculated between genotypes using the unweighted pair group method with arithmetic averages (UPGMA). This matrix was used to construct the phylogenetic tree according to the Euclidean similarity index using the PAST software Version 1.91 [32]. The performance of SCoT and ISSR primers was measured by calculating the polymorphic information content (PIC). The PIC value was calculated for each locus according to Roldán-Ruiz et al. [33] as follows: PICi = 2fi (1 − fi), where PICi = PIC of the locus i, fi = frequency of the amplified fragments (band present), and (1 − fi) = frequency of non-amplified fragments (band absent). The PIC of each primer was calculated using average PIC values from all loci of each primer. The ClustVis tool [34] was used for the development of the heatmap. It was constructed using the data matrix of SCoT and ISSR analysis to map the highest and lowest dataset values with a color gradient.

#### Genomic template stability

The genomic template stability (GTS) is a DNA stability qualitative measure. GTS reflects the change in the banding patterns of SCoT and ISSR generated by crude oil-treated and control plants. The average GTS was calculated as a percentage of the control according to the formula of Liu et al. [35] as follows: GTS% = **[**1 − (*a*/*n*)**]** × 100, where *a* = the average number of polymorphic bands in crude oil-treated plants, and *n* = the average number of all bands in control plants.

#### Data analysis

The data were statistically analyzed by using SPSS 18.0 for Windows. The obtained values are expressed as mean±SE. The different treatments were compared using the one-way ANOVA and Duncan’s multiple range test (*p* ≤ 0.05).

## Results

### TPH degradation

**Fig. 1:**
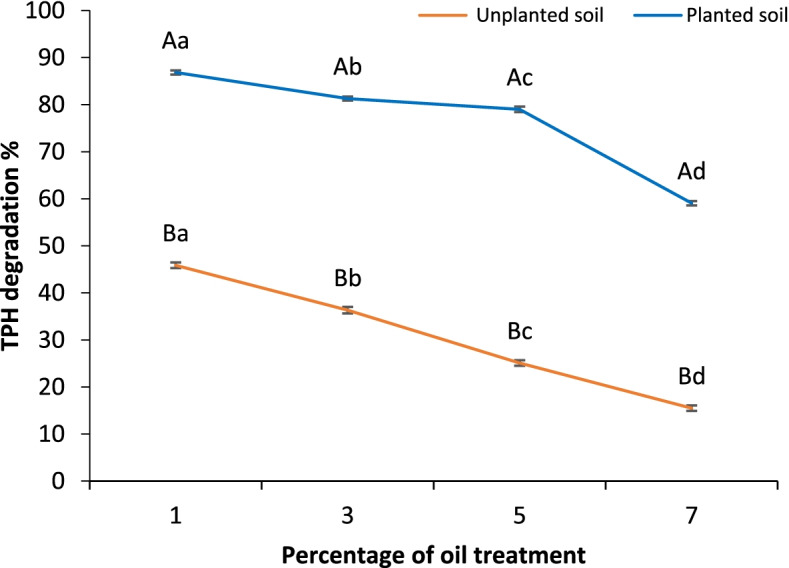
TPH degradation percentage of soil treated with different levels of crude oil of 0% (control), 1%, 3%, 5%, and 7% and planted with *Vinca rosea* compared to unplanted polluted soil after a 5-month experimental period. Different uppercase letters indicate significant differences between the planted and unplanted treatments at each oil concentration, and different lowercase letters indicate significant differences among different soil treatments at *p* ≤ 0.05. Values are expressed as the mean ± SE, *n* = 3

The degradation percentage of TPH (total petroleum hydrocarbons) in soils planted with *V. rosea* and the corresponding unplanted controls varied based on the crude oil treatment levels after a 5-month growth period (Fig. 1). The degradation percentage in the presence of plants ranged from 86.83 ± 0.44% to 59.05 ± 0.45% in soils treated with 1 and 7% oil: soil by weight, respectively. In the corresponding unplanted controls, the TPH degradation percentage ranged from 45.87 ± 0.59% and 15.50 ± 0.58% in 1 and 7% treatments, respectively. The TPH degradation percentage in planted soils declined with the increase in crude oil treatment level. The same trend was observed for unplanted soils. The obtained results indicate that the percentages of TPH degradation at pollution levels of 1 to 5% were higher than the degradation at the 7% level.

### Seed germination and plant growth

**Fig. 2:**
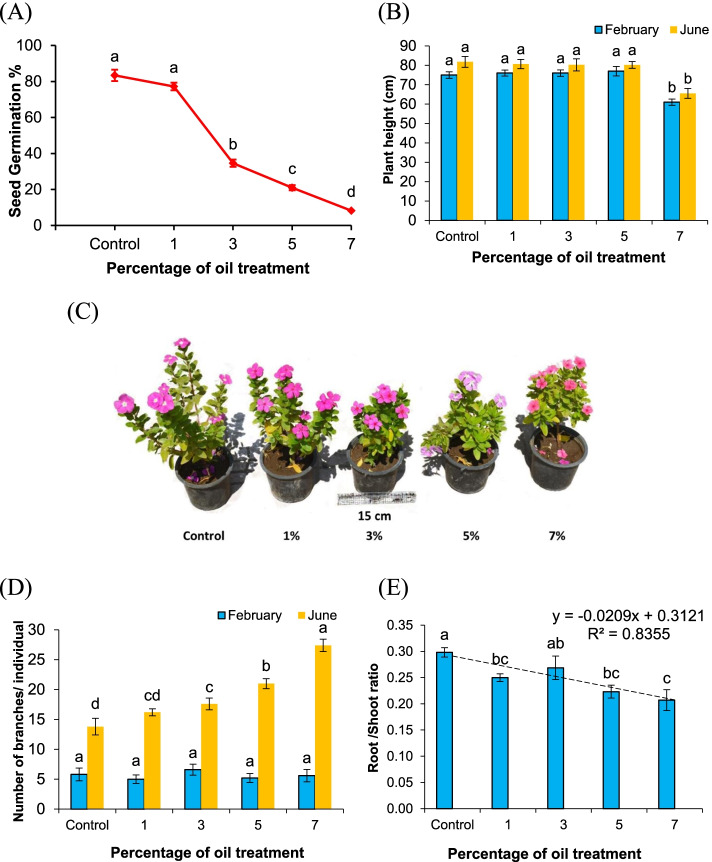
Seed germination and plant growth of *Vinca rosea* raised in soils treated with different crude oil levels of 0% (control), 1%, 3%, 5%, and 7%. **A** Seed germination percentage, **B** plant height, **C** *Vinca rosea* growth after a 5-month growth period, **D** number of branches per individual, and **E** root/shoot ratio. Different letters indicate significant differences among different soil treatments at *p* ≤ 0.05. Values are expressed as mean ± SE (*n*= 5). The dashed line in 1D is the linear regression of root/shoot ratio against the percentage of crude oil treatment

A low level of crude oil treatment of 1% had a non-significant effect on the germination of *V. rosea* seeds. There was a progressive decline in seed germination percentage with the increase in crude oil percentage in the soil (Fig. 2A). The germination percentage of seeds in soil treated with 1% crude oil reached 77.2 ± 3.15%, which was not significantly different from the germination of control seeds (83.40 ± 2.36%). The most affected seeds, for which the lowest germination percentage was 8.2 ± 1.43%, were found in 7% crude oil-treated soils. The seed germination in polluted soils decreased with an increased pollution level up to 7%; above this concentration, germination failed.

The height of plants raised under crude oil pollution levels of 1, 3, and 5% after 1- and 5-month growth periods was not significantly different to the control (Fig. 2B, C). After a 1-month growth period, the control plants reached a height of 75 ± 3.55 cm, but there was no significant difference in plants raised under crude oil treatment levels from 1 to 5%. The plant height significantly decreased to 61 ± 2.92 cm at a 7% pollution level. After a 5-month growth period, the plant height followed a similar trend, with the values at a 7% treatment level reaching 81.78 ± 2.79 cm in the control plants and 65.52 ± 2.54 cm in the treated plants.

The number of branches in plants raised under crude oil pollution remained unaffected over a 1-month growth period, but significantly increased with an increase in crude oil soil pollution over 5 months of growth (Fig. 2C, D). In the control treatment, the number of branches was 13.80 ± 1.39 per individual. The highest number of plant branches reached 27.40 ± 1.03 per individual for the 7% crude oil treatment.

The root/shoot ratio after a 5-month growth period decreased with the increase in oil pollution levels in the soil (Fig. 2E). The values were 0.30 ± 0.01 in the control plants and lower in plants raised under the different oil treatments. The lowest value attained was 0.21 ± 0.02 in plants raised under a 7% crude oil pollution level in the soil.

In general, as the crude oil treatment levels in the soil increased, the seed germination, plant height, and root/shoot ratio decreased, while the number of branches per individual increased.

#### Phytomass allocation

**Fig. 3:**
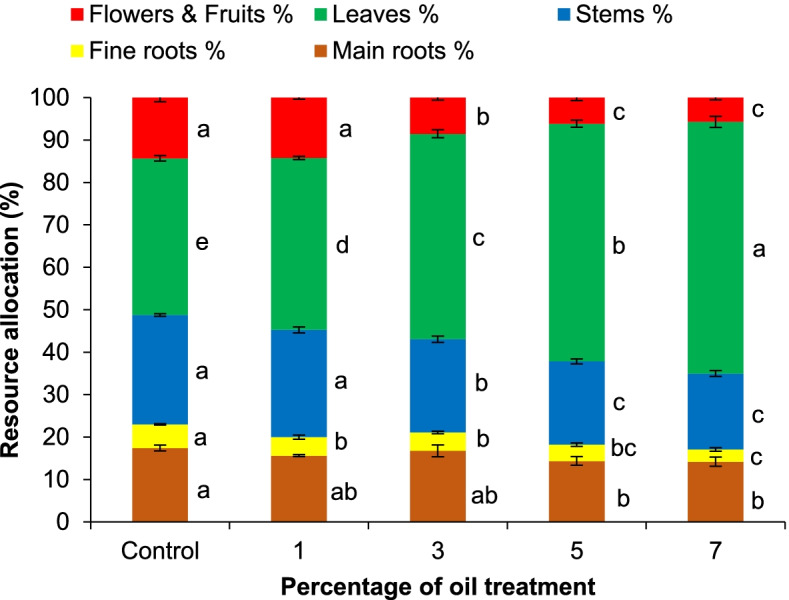
Phytomass allocation of *Vinca rosea* plants raised under different crude oil treatment levels of 0% (control), 1%, 3%, 5%, and 7% at the end of the 5-month experimental time. Different letters indicate significant differences between different treatments at *p* ≤ 0.05. Values are expressed as mean ± SE (*n* = 5)

The phytomass allocation in control plants was 17.41% ± 0.68% to the main taproots, 5.55% ± 0.18% to the fine roots, 25.81% ± 0.28% to the stems, 36.90% ± 0.66% to the leaves, and 14.33% ± 0.98% to the flowers and fruits (Fig. 3). Under 1% oil treatment, the percentage of phytomass allocated to the fine roots decreased to 4.37% ± 0.47%, while values allocated to the leaves increased to 40.53% ±0.4%. The values allocated to the fine roots, stems, and flowers and fruits decreased to 4.32% ± 0.3%, 22% ± 0.76%, and 8.53% ± 0.57%, respectively, under 3% oil treatment. Alternatively, the allocation to leaves under 3% oil treatment increased to 48.40% ± 0.95%. For plants raised under 5% oil treatment, the allocated resources decreased to 14.34% ± 1.02%, 3.86% ± 0.4%, 19.63% ± 0.6%, and 6.15% ± 0.7% in the main roots, fine roots, stems, and flowers and fruits, respectively, whereas values increased in the leaves to 56.01% ± 0.82%. The resource allocation in the plants treated with 7% oil showed a similar trend to the treatment with 5% oil, whereas values decreased in the main roots, fine roots, stems, and flowers and fruits to 14.17% ± 1.07%, 2.9% ± 0.43%, 17.89% ± 0.69%, and 5.69% ± 0.53%, respectively, while vaules in the leaves increased to 59.35% ± 1.3%. As the increase in crude oil treatment levels in the soil increased from 1 to 7%, the overall trends in phytomass allocation to different plant organs were lower in the roots, stems, and reproductive organs, but increased in the leaves.

### Chlorophylls and carotenoid content

**Fig. 4:**
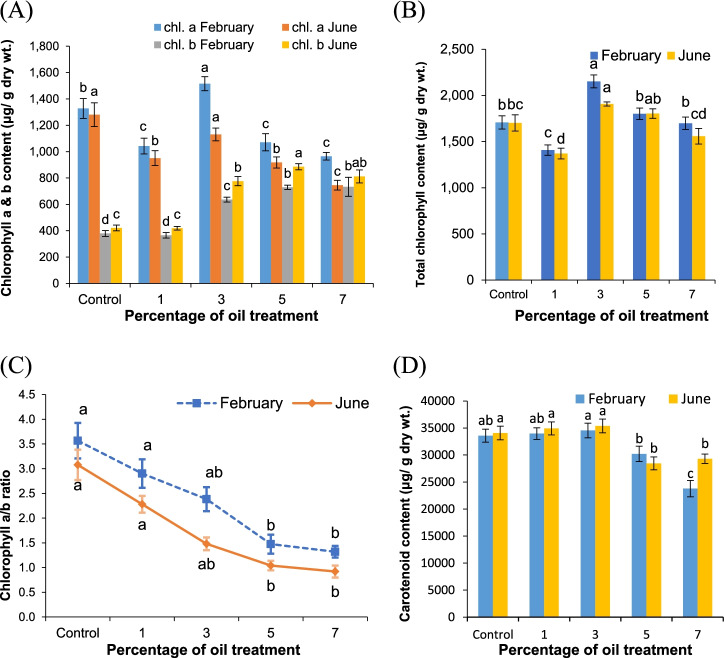
Chlorophyll and total carotenoid contents of *Vinca rosea* plants raised under different crude oil treatment levels of 0% (control), 1%, 3%, 5%, and 7% after 1 month and 5 months of growth. **A** Chlorophyll a and b content, **B** total chlorophyll content, **C** chlorophyll a/b ratio, and **D** total carotenoid content. Different letters indicate significant differences between different treatments at *p* ≤ 0.05. Values are expressed as mean ± SE (*n* = 5)

The data variations in *V. rosea* leaf chlorophyll content in response to soil treatment with crude oil are shown in Fig. 4A. The chlorophyll a content decreased as the crude oil treatment levels increased in the soil, with values ranging from 1327.80 ± 75.28 μg/g dry weight in the control group to 965.20 ± 28.74 μg/g dry weight at 7% oil treatment after a 1-month growth period and from 1280.40 ± 90.14 to 745.60 ± 37.11 μg/g dry weight after a 5-month growth period. As for chlorophyll b content, the values were nonuniformly increased under the different crude oil treatments and attained 379.40 ± 22.35 μg/g dry weight in the control to 733.40 ± 72.64 μg/g dry weight after 1-month growth, while after 5 months, the values varied between 421.20 ± 21.53 μg/g dry weight in the control and 812.00 ± 49.31 μg/g dry weight.

The total chlorophyll content attained the maximum values in the plants raised under 3% crude oil treatment and amounted to 2151.80 ± 69.56 μg/g dry weight, after 1 month of growth and 1907.00 ± 21.98 μg/g dry weight after a 5-month growth period (Fig. 4B). The minimum chlorophyll a + b values under 1% crude oil treatment reached 1407.60 ± 56.18 μg/g dry weight after 1 month and 1370.20 ± 57.95 μg/g dry weight after 5 months.

The chlorophyll a/b ratio was higher than unity with decreasing values from 3.57 ± 0.36 in the control and 1.32 ± 0.12 under 7% crude oil treatment after 1-month growth (Fig. 4C). Similarly, the ratio decreased with an increase in oil pollution level after 5 months, to 3.08 ± 0.31 in the control and 0.92 ± 0.12 under 7% crude oil treatment.

In contrast to what occurs for chlorophylls, the total carotenoids were increased in the control after 1- and 5-month growth periods under crude oil treatments of 1 and 3%, but decreased from the control under 5 and 7% treatments (Fig. 4D). The maximum value of carotenoids was obtained under 3% crude oil treatment, of 34,547 ± 1351 and 35,383 ± 1271 μg/g dry weight after 1- and 5-month growth periods, respectively. The lowest values were 23,780 ± 1519 μg/g dry weight after 1 month under a 7% treatment level and 28,455 ± 1196 μg/g dry weight under a 5% treatment level over the experimental period.

As shown in Fig. 4, chlorophyll a values decreased, and chlorophyll b values increased, as reflected in the decreased chlorophyll a/b ratio with the increased pollution levels of crude oil in the soil. The carotenoid content increased at low crude oil levels of 1 and 3% but decreased at 5 and 7% levels of soil pollution.

### Content of tannins, phenolics, flavonoids and antioxidant capacity

**Fig. 5:**
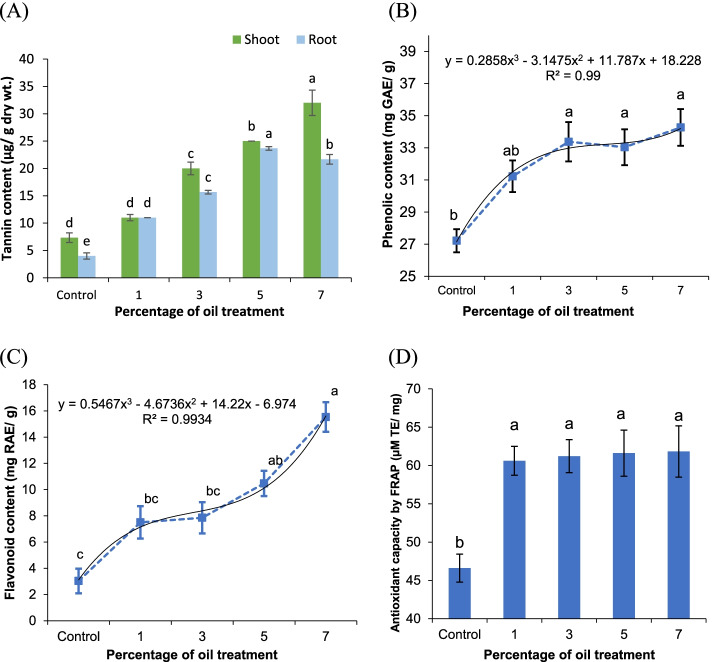
Tannins, phenolics, flavonoid content, and antioxidant capacity by FRAP of *Vinca rosea* plants raised under different crude oil treatment levels of 0% (control), 1%, 3%, 5%, and 7% at the end of a 5-month experimental time. **A** Tannin content of shoot and root, **B** phenolic content in shoots, **C** flavonoid content in shoots, and **D** antioxidant capacity. The shown curves **A** and **B** are the polynomial regression (order 3) of flavonoid and phenolic content against the percentage of crude oil treatment. Different letters indicate significant differences between different treatments at *p* ≤ 0.05. Values are expressed as mean ± SE (*n* = 3)

The content of tannins in shoots and roots of *V. rosea* was estimated at the end of the experiment in June (Fig. 5A). The tannin values in the shoot system increased from 7 ± 0.88 μg/g dry weight in the control to 32 ± 2.31 μg/g dry weight under the 7% crude oil treatment level. As for the root system, the values increased from 4.00 ± 0.58 μg/g dry weight in the control to 23.66 ± 0.33 μg/g dry weight at the 5% treatment level, and then decreased to 21.66 ± 0.88 μg/g dry weight at the 7% treatment level. As shown, the tannin content was up to five times higher in the shoot and up to six times higher in the roots of plants raised under crude oil treatment.

The phenolic content increased with the increasing crude oil treatment level in the soil (Fig. 5B). The values reached 27.22 ± 0.72 mg gallic acid equivalents (GAE)/g in the shoots of control plants and increased to 34.27 ± 1.15 mg GAE/g extract in plants raised under the high level (7%) of crude oil treatment. At low levels of crude oil soil treatment (up to 3%), higher concentrations of phenolics accumulated under 5 and 7% treatments.

The flavonoid content increased with an increased level of crude oil treatment (Fig. 5C). The lowest flavonoid content was observed in the control plants, of 3.03 ± 0.94 mg rutin acid equivalents (RAE)/g, and increased to reach the lowest value of 15.53 ± 1.13 mg (RAE)/g in plants raised under 7% crude oil treatment.

The ferric reducing ability of plasma (FRAP), a measurement of the antioxidant capacity, was increased in plant shoots raised under crude oil treatment 1, 3, 5, and 7% in comparison to the control (Fig. 5D). The values reached 46.61 ± 1.825 μM Trolox equivalents (TE)/mg in the control sample and ranged from 60.61 ± 1.88 to 61.83 ± 3.345 μM TE/mg in the plants raised in soils treated with crude oil. The overall effect of soil pollution with crude oil demonstrated the increased content of tannins, phenolics, and flavonoids and antioxidant capacity with the increased crude oil treatment.

### Genotoxicity

**Fig. 6:**
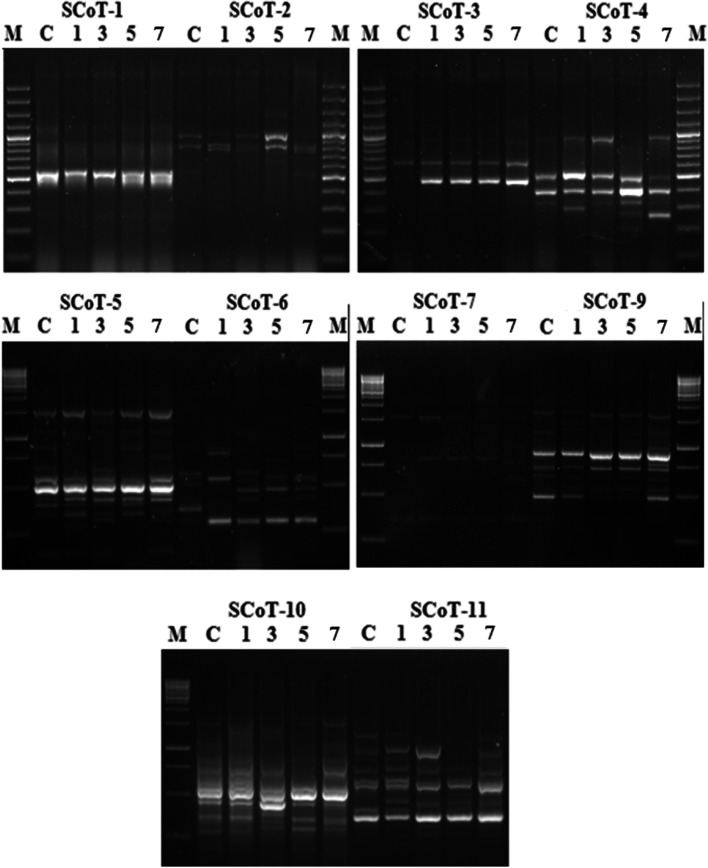
SCoT patterns of *Vinca rosea* plants raised under crude oil treatment levels of 0% (control, C), 1%, 3%, 5%, and 7% for a 5-month growth period and 100-bp ladder (M). The analysis was performed using the 10 primers: SCoT-1, SCoT-2, SCoT-3, SCoT-4, SCoT-5, SCoT-6, SCoT-7, SCoT-9, SCoT-10, and SCoT-11

**Fig. 7:**
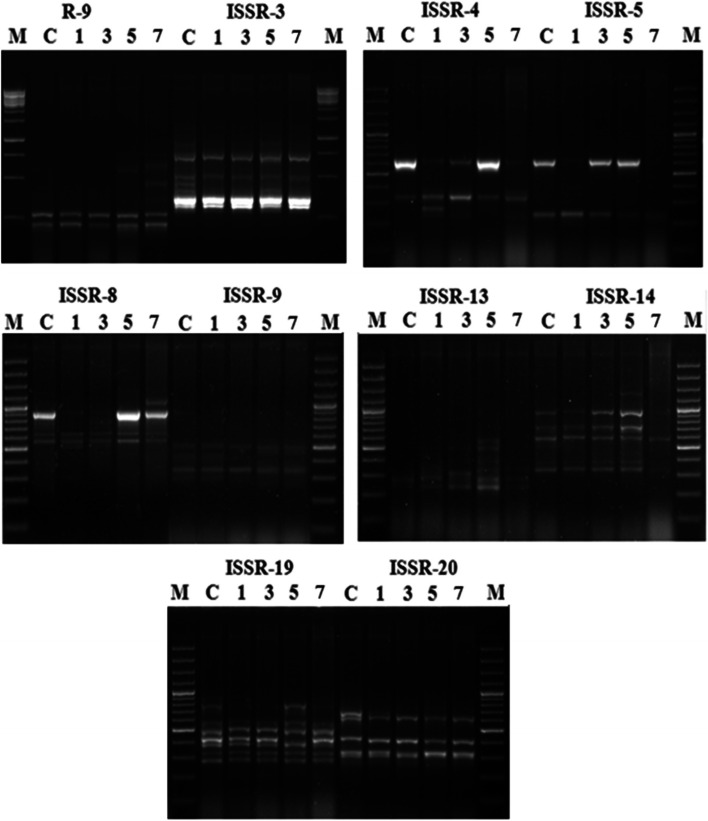
ISSR patterns of *Vinca rosea* plants raised under crude oil treatment levels of 0% (control, C), 1%, 3%, 5%, and 7% for a 5-month growth period and 100-bp ladder (M). The analysis was performed using the 10 primers: ISSR-4, ISSR-5, ISSR-8, ISSR-9, ISSR-13, ISSR-14, ISSR-19, ISSR-20, R-9, and ISSR-3

The genotoxic effect of crude oil on *V. rosea* plants was tested using SCoT and ISSR molecular markers. The primers produced amplification profiles, and the reproducible patterns were screened for the presence of polymorphism. The results obtained from the ISSR and SCoT analyses showed molecular variations between the banding profiles of control plants and plants raised under different crude oil treatments (Figs. 6 and 7).

In total, SCoT amplifications produced 107 bands across the five crude oil treatments (0, 1, 3, 5, and 7% crude oil treatment), an average of 10.7 bands/primer and a size range of 200–2000 bp. Of the 107 bands, 57 (53.3%) were polymorphic (Table 4, Fig. 6). The number of amplified fragments for each of the used primers varied from five bands using SCoT-3 to 16 bands using SCoT-5. One of the 10 primers, SCoT-1, did not demonstrate differences in banding profile between the control sample and the experimental crude oil treatments. The remaining primers amplified polymorphic bands with different polymorphism percentages, which ranged from 25% produced by SCoT-9 to 93% using SCoT-6, with a 52.1% average level of polymorphism. The calculated frequency of primers that produced polymorphic bands ranged from 0.4 using SCoT-6 to 0.8 using SCoT-9 and SCoT-11.

**Table 4:** Characteristics of SCoT and ISSR banding profiles produced from experimental groups of *Vinca rosea* plants raised under different crude oil treatment levels of 0% (control), 1%, 3%, 5%, and 7%

SCoT primer name	Size range (bp)	TB	MB	PB	% P	F	PIC	ISSR primer name	Size range (bp)	TB	MB	PB	% P	F	PIC
**SCoT-1**	270–1100	6	6	0	0	1	0.0	**ISSR-3**	260–1200	10	7	3	30	0.8	0.28
**SCoT-2**	520–1400	7	2	5	71	0.6	0.35	**ISSR-4**	240–650	7	3	4	57	0.7	0.34
**SCoT-3**	500–1300	5	2	3	60	0.7	0.35	**ISSR-5**	230–650	5	3	2	40	0.8	0.29
**SCoT-4**	200–1000	10	5	5	50	0.7	0.33	**ISSR-8**	350–1200	9	5	4	44	0.7	0.30
**SCoT-5**	200–1500	16	6	10	63	0.6	0.35	**ISSR-9**	280–550	8	3	5	63	0.6	0.35
**SCoT-6**	300–1300	15	1	14	93	0.4	0.36	**ISSR-13**	240–600	7	2	5	71	0.6	0.35
**SCoT-7**	350–2000	7	3	4	57	0.6	0.36	**ISSR-14**	330–1400	8	5	2	38	0.8	0.22
**SCoT-9**	350–1600	12	9	3	25	0.8	0.23	**ISSR-19**	200–900	10	7	3	30	0.9	0.21
**SCoT-10**	300–1500	14	8	4	42	0.7	0.28	**ISSR-20**	270–700	7	4	3	43	0.8	0.29
**SCoT-11**	250–1500	15	6	9	60	0.8	0.29	**R-9**	200–750	7	2	5	71	0.5	0.36
**Total**		**107**	**48**	**57**	**-**	**-**	**-**	**Total**		**78**	**41**	**36**	**-**	**-**	**-**
**Means**		**10.7**	**4.8**	**5.7**	**52.1**	**0.69**	**0.29**	**Means**		**7.8**	**4.1**	**3.6**	**48.7**	**0.72**	**0.30**

Molecular size range of amplified fragments in bp (size range), total number of bands amplified (TB), monomorphic bands (MB), polymorphic bands (PB), percentage of polymorphism (%P), frequency (F), and polymorphism information content (PIC)

The ISSR primers produced a total of 78 bands, with an average of 7.8 bands per primer. The bands produced ranged between 200 and 1400 bp. Of the 78 bands, 36 (64.2%) were polymorphic (Table 4, Fig. 7). The 10 primers produced polymorphic bands with different percentages of polymorphism that ranged from 71% using ISSR-13 and R-9 primers to 30% using ISSR-3 and ISSR-19 primers with an average of 48.7%. The number of produced fragments varied from 5 using the ISSR-5 primer to 10 using ISSR-3 and ISSR-19. The highest frequency was 0.9 with the ISSR-19 primer and the lowest frequency reached 0.5 with the R-9 primer.

The values of polymorphic information content (PIC) were calculated for each primer of both SCoT and ISSR, considering all bands of each primer. The range of PIC in SCoT analysis ranged from 0 for SCoT-1 to 0.36 for SCoT-6 and SCoT-7, with an average of 0.29 (Table 4). For ISSR primers, the PIC varied from 0.21 for ISSR-19 to 0.36 for R-9, with an average PIC value of 0.30.

### Genetic similarity

The calculated genetic similarity from SCoT and ISSR profiles revealed that the similarity between control *V. rosea* plants and those raised under crude oil treatments decreased with an increase in the treatment level (Supplementary Table 1A). For the SCoT primers, the highest similarity to control plants reached 0.91 for the plants raised under 1% oil treatment and decreased to 0.75 for plants raised under 7% oil treatment. In the case of ISSR profiles, the genetic similarity was 0.95 between the control plants and those raised under 1% oil treatment and 0.72 for plants raised under 7% treatment (Supplementary Table 1B). The genetic similarity, in general, among plants raised in soils polluted with different levels of crude oil decreased with the increased level of soil pollution.

**Fig. 8:**
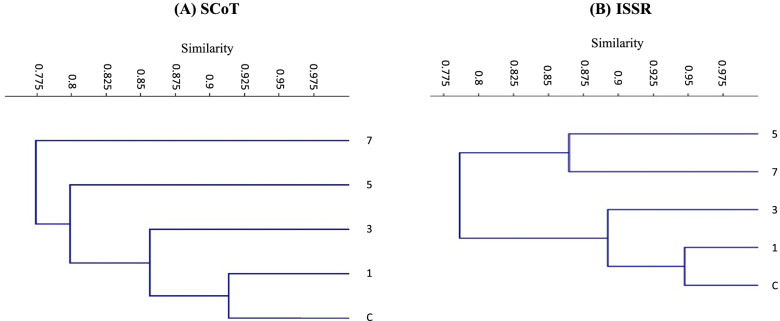
The constructed dendrogram based on SCoT analysis (**A**) and ISSR analysis (**B**). The two dendrograms show the genetic distance between control (**c**) and plants raised in soils treated with different crude oil levels of 1%, 3%, 5%, and 7% for a 5-month growth period

**Fig. 9:**
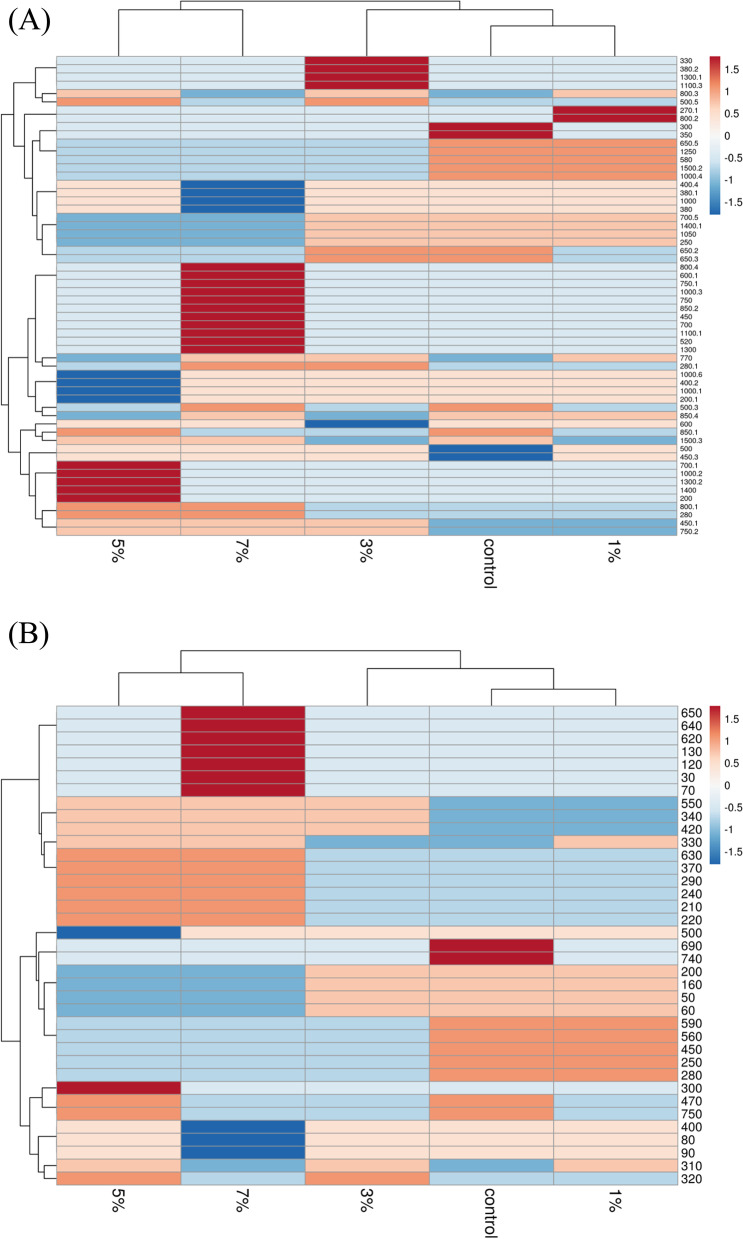
Heatmap analysis for *Vinca rosea* plants raised under different crude oil treatment levels for a 5-month growth period as based on SCoT profiling datasets (**A**) and ISSR profiling datasets (**B**). The different treatment groups, control (0%), 1%, 3%, 5%, and 7%, are in columns and the obtained bands are in rows

The constructed dendrograms, based on SCoT and ISSR analysis, showed a treatment-dependent trend where the plants raised under low pollution levels of crude oil were grouped at a closer distance to the untreated control plants. For the SCoT dendrogram (Fig. 8A), two main groups are separated, one cluster for plants raised under a 7% crude oil treatment level. The second group was divided into subgroups, one subgroup for plants raised under 5% crude oil treatment and the second comprised the two remaining subsubgroups. Alternatively, in Fig. 8B, the produced dendrogram, as based on the ISSR data, comprised two main groups: the first includes the plants raised under the two crude oil treatments, 5 and 7%, and the second group included the control and the plants raised under 1 and 3% crude oil treatments. The heatmap provides a more precise visualization of the genetic similarity among the plants in different treatment groups using SCoT and ISSR banding profiles (Fig. 9A, B). The red color in the heat map reveals a high level of representation, blue represents a low level of representation, and the decreasing/increasing color intensity is directly proportionate to the studied character value.

### Genomic DNA template stability

The polymorphism in SCoT and ISSR patterns in *V. rosea* plants raised under crude oil treatments are presented based on genomic template stability (GTS). The calculated polymorphism based on the appearance and disappearance of bands is shown in Supplementary Table 2.

The generated data by SCoT analysis showed a total of 75 bands in the control set. The number of bands ranged from 2, produced using SCoT-3, to 12, using SCoT-10 and SCoT-11. Of the ten primers used, nine primers amplified stable and specific patterns with a significant change in the number of produced fragments between the control plants and treated plants. The plants raised under crude oil treatments of 1, 3, 5, and 7% showed different banding profiles compared to the control, with the appearance or disappearance of bands in the profiles. The appearance of new bands increased as the crude oil treatment percentage increased. The disappearance of bands increased as the crude oil treatment percentage increased.

The data generated from ISSR analysis revealed a total of 58 bands in the control profile. The number of bands per primer varied from 2 for ISSR-13 to 10 for ISSR-3 and ISSR-19. The 10 primers produced stable and specific profiles, which showed a significant change in the number of bands between plants raised under crude oil treatments and control plants. The *V. rosea* plants raised under the lowest crude oil treatment (1%) had two new bands; under the highest treatment (7%), 16 new bands appeared. The number of bands that disappeared ranged from 4 in the profiles of plants raised under 1% crude oil treatment to 15 in the profiles of plants raised under 7% crude oil treatment.

**Fig. 10:**
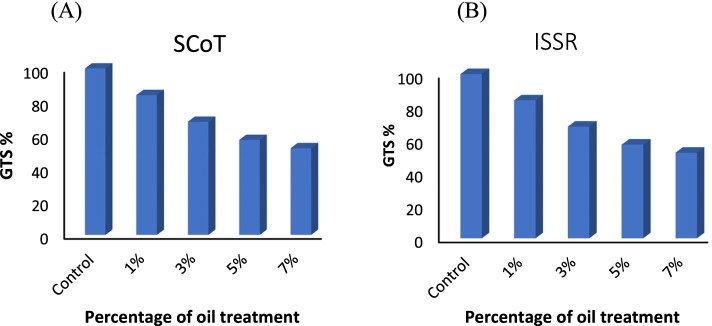
Genomic DNA template stability (GTS) based on the appearance and disappearance of bands in SCoT and ISSR profiles of *Vinca rosea* plants raised under different crude oil treatment levels of 0% (control), 1%, 3%, 5%, and 7% for a 5-month growth period with the control. **A** GTS based on SCoT profiles and **B** GTS based on ISSR profiles

The GTS was based on the disappearance and appearance of bands in SCoT and ISSR profiles, as shown in Fig. 10A, B. The two profiles of SCoT and ISSR had similar trend of stability reduction with an increase of crude oil treatment level. SCoT profiles revealed a GTS reduction range of 84 to 52%, while ISSR profiles showed GTS reduction range of 90 to 57% in plants raised under 1, 3, 5, and 7% crude oil treatment.

## Discussion

Terrestrial and aquatic ecosystems in oil-producing countries are subject to variant pollution levels, which affect people’s lives and biodiversity owing to the accumulation of toxic pollutants. Different technologies have been tested to combat pollution and remediate polluted ecosystems [36]. As a green technology, phytoremediation is among the safest and least expensive methods that are used to clean up polluted soils and water. This study evaluates the potential use of the ornamental plant *V. rosea* L. as a phytoremediator of crude oil-polluted soil and assesses the induced genotoxicity by using SCoT and ISSR molecular markers. The results of this study demonstrated the significance of *V. rosea* in crude oil phytodegradation at low pollution levels of up to 5%, which affected the plant’s functional traits and molecular variations.

Crude oil degradation was significantly enhanced in planted soils, particularly at low crude oil treatment levels (up to 5%) in comparison to the unplanted controls. As shown by Nie et al. [37], a higher level of crude oil might have a toxic effect on plants and the surrounding microflora, which would subsequently cause a reduction in TPH degradation in soils. Petroleum hydrocarbons can be degraded chemically, physically, or biologically. Crude oil degradation in unplanted soil could occur as a result of adsorption, leaching, evaporation, oxidation in light exposure, and biodegradation [38]. The significant enhancement of TPH degradation by *V. rosea* might be attributable to its well-developed rooting system, which can provide a wider surface area for the activation of oil-degrading microflora. The report of Hou et al. [39] indicated that the rooting system is an essential factor required for TPH degradation, where some soil microbes can mineralize root exudates while being used as growth substrates, which can further act as co-metabolites for persistent petroleum hydrocarbon degradation [40].

The effect of crude oil pollution in soil on plants resulted in disturbance of the morphological and physicochemical traits, decreased seed germination, growth, and biomass production, as well as reduced availability of oxygen, water, and nutrients [41]. These effects disturb the food web, which has effects on human food chain and health. The crude oil pollution of soils causes water deficits, anaerobic conditions, deficiency of essential elements, oxidative stress, protein synthesis inhibition, and DNA damage leading to cell death [42–44].

In response to soil pollution by different levels of crude oil within the range 1–7%, *V. rosea* showed the ability to regulate seed germination, growth, and phytomass allocation according to crude oil treatment percentage. The decreased seed germination results from the penetration of petroleum hydrocarbons into the seeds, causing metabolic reactions to be altered and preventing water and oxygen uptake [45]. The plant shoot height and number of branches were significantly affected by a crude oil treatment level of 7% after a 5-month growth period: the height attained was the lowest value of 65.52 ± 2.54 cm, and the number of branches was the highest per individual of 27.40±1.03 branches: i.e., branching density increased owing to the stresses applied by crude oil. This seems to be attributed to the stress produced by crude oil pollution and its degradation products [2, 3, 8], indicating that not only does crude oil affect plant growth but also that the level of pollution has an effect; plants may adapt at low levels, whereas growth is interrupted at higher levels of pollution [8]. From this perspective, one may infer that plant growth may indicate the level of soil pollution as a phytoindicator / bioindicator [46, 47].

Growth and branching patterns are important functional traits for understanding how plants respond to pollution [48]. The association of increased branching priority with decreased height balances the allocation of resources, enabling plants to grow and produce more leaves under pollution stress conditions [49, 50]. The increased branching in *V. rosea* optimizes phytoremediation to compensate for the low plant growth and height triggered by pollution stress [50].

The phytomass allocation to different plant organs is regulated in a strategic exchange manner to maximize its adaptive response to environmental stresses [51]. Therefore, under low crude oil pollution levels, *V. rosea* allocated lower amounts of phytomass to leaves, but higher amounts to stems, flowers and fruits, and roots. However, the opposite was true at high levels of soil pollution, which was confirmed in some other studies [42] as an adaptive strategy to maximize its photosynthetic activity under stress conditions. This “trade-off” tactic in phytomass allocation is important for *V. rosea* as a phytoremediator to support its survival under crude oil stress. The low values of root/shoot phytomass ratio, of less than 0.30 under soil treatment with crude oil pollution, may indicate that root phytomass is synchronized with the plant’s ability to tolerate the adverse effects of pollution stress. This is confirmed by other studies on different environmental stresses [42, 52].

The major impact of crude oil is a decrease in the synthesis of chlorophyll pigment owing to the effect of high molecular weight aromatic, aliphatic, and organic compounds that inhibit the enzymes required for chlorophyll synthesis [53]. In addition, the decrease in total chlorophyll content in the leaves may occur due to the mixing of oil constituents in the soil, which increases the degradation of chlorophyll. The photosynthetic pigment content of *V. rosea* demonstrated perturbations due to crude oil stress [2, 54] as a result of the phytotoxic effects of crude oil and its degradation products. This seems to be attributed to the degradation of chlorophyll and photosynthetic pigments due to the stress produced by crude oil soil pollution [47, 55].

The content of chlorophyll a and b displayed opposing trends change in response to the level of crude oil treatment. Chlorophyll a values decreased with an increase in crude oil treatment, although the opposite was true for chlorophyll b, as demonstrated by the changes in chlorophyll a/b ratio. The decrease in chlorophyll a/b ratio with the increase in crude oil treatment is considered an adaptive strategy expand the range of light harvesting through different light spectra [2, 56].

The carotenoid content was significantly decreased at crude oil treatment levels of 5 and 7%. Therefore, their role in the mitigation of oxidative stresses was reduced [57, 58]. The decreased amounts of carotenoids associated with increased genotoxicity in response to crude oil pollution of the soil may be attributed to its oxidation under environmental stresses [59]. This demonstrates the possible role of carotenoids in the preservation of the DNA in the plant cell from oxidative damage. The increase in carotenoid content with the decrease in chlorophyll a/b ratio suggests resistance to degradation as carotenoids are relatively stable and protective pigments.

Many metals are essential for the functioning of metabolic pathways in green plants. However, excessive amounts of heavy metals interfere with the cellular metabolism and induce toxicity due to the inactivation of biological activities [60]. Among the most toxic metals to higher plants are the metal ions of Cd^2+^, Pb^2+^, Pb^4+^ Zn^2+^, Cu^2+^, and Co^2+^, particularly when occurring at high concentrations, owing to the presence of environmental contaminants, including petroleum oil, which lead to the production of free radicals, oxidative stress-causing reactive oxygen species (ROS) [61], disruption of plant metabolism, decreased chlorophyll a/b ratio, and DNA damage [2, 60].

Many studies have confirmed the role of tannins in plant cells as protective and defensive compounds [56, 62] against both biotic and abiotic stresses. Increased tannin content in *V. rosea* shoots and roots with increased crude oil soil pollution is viewed as a defense mechanism to protect plant organs from pollution or environmental stresses [56, 63].

The plant system has several oxidative reactions against toxicity, which are essential for the viability of cells and to mitigate oxidative stress. The crude oil increased the total antioxidant capacity and the antioxidant metabolites, phenolics, and flavonoids, which are the main contributors to FRAP changes. Phenolics and flavonoids are biologically active compounds that play an important role as stress-preventing agents in plant resistance to adverse environments [64, 43]. Among the many functions of phenolics and flavonoids, they help plants to live on soils that are rich in toxic metals [65] and assist plant growth on poor soils when they are released into the rhizosphere to improve the plant-microbe partnership in the root active zone, which may speed up the degradation of organic and complex compounds in the soil [66]. According to Da Silva and Maranho [67], Moubasher et al. [8] and Arslan et al. [68], the phytoremediation of crude oil-polluted soils is usually facilitated by plant–microorganism interactions in the rhizosphere zone. This synergistic degradation maximizes the phytoremediation of crude oil compounds. The significant increase or maintenance of phenolic and flavonoid levels with increased oil pollution has established their potential role as biochemical markers of stress responses [65]. It is reported that phenolics and flavonoids are important antioxidants that may participate in protecting plants from the oxidative damage caused by different environmental stresses and protect the plant photosynthetic machinery from stress [69].

The prolonged and persistent exposure of living organisms to environmental contaminants may cause anomalies at the molecular level [70], where petroleum hydrocarbons are identified as carcinogenic and mutagenic pollutants [71]. Although DNA molecular markers are mostly used for the detection of variations in the studies of phylogeny, genetic diversity, genome mapping, gene tagging, evolution, and molecular ecology [72, 73], DNA damage can be monitored accurately by using molecular markers [72]. SCoT and ISSR markers are considered more reliable and accurate tools than the other DNA molecular markers [13]. The SCoT analysis detects the polymorphism in the coding sequence, allowing it to be correlated to functional genes, whereas the ISSR analysis detects the microsatellite polymorphism, which is not necessarily related to functional genes [14].

In the present study, the markers revealed discriminative DNA patterns of control and crude-oil-treated *V. rosea* plants. The characteristics of the banding profiles produced by SCoT and ISSR markers showed a high level of genetic polymorphism in the control and the four *V. rosea* experimental groups grown in crude oil soil with treatment levels of 1, 3, 5, and 7%. The number of produced bands per primer is an important parameter indicating the marker efficiency and informativeness in the analysis of genetic variation [15]. The SCoT markers produced a higher number of bands per primer (10.7) than ISSR markers (7.8). Similarly, Amom et al. [74] demonstrated that the number of bands produced by SCoT markers (13.8) was higher than the number of bands produced by ISSR markers (11.5) in their study of the genetic relationship between five bamboo species in North-East India. The presence of more bands per primer (9.5) for SCoT markers was also reported by Pakseresht et al. [75], who observed 4.75 bands per primer for ISSR markers in their study on the genetic diversity of 40 genotypes of chickpea in Iran. Amirmoradi et al. [76] demonstrated a higher number of scoreable bands per primer (12.4) for the SCoT markers than for the ISSR markers (11.5) in the study of the genetic relationships among 38 accessions of 8 annual Cicer species. The SCoT markers also produced a higher number of bands (8.7) than the ISSR markers (6.8) in the genetic comparison analysis of 24 tetraploid potato varieties [13]. As reported by Luo et al. [15], more scoreable bands per primer were found for the SCoT markers when compared with the ISSR markers (8.8 versus 7 bands per primer) in the analysis of 23 mango accessions from Guangxi, China.

The ability of a molecular marker to generate polymorphic bands and its PIC value can also be used to assess its ability to discriminate genetic variation [77]. The determined polymorphism percentage for SCoT and ISSR markers indicated their efficiency in distinguishing the genetic variation that occurred in response to soil pollution with crude oil. The polymorphism percentage detected by the SCoT marker (52.1%) was higher than that detected by the ISSR marker (48.7%). A higher percentage of polymorphism was similarly found for SCoT markers than that of ISSR markers in other studies on other plant species [76, 78]. The PIC values for SCoT primers that produced polymorphic bands varied from 0.23 to 0.36 with an average of 0.32, while for ISSR the values ranged from 0.21 to 0.36 with an average of 0.30. Higher PIC average values than those of ISSR markers have been reported in other plant species [74, 79].

The results obtained in this study inferred that SCoT and ISSR markers can effectively determine the genotoxic effect caused by crude oil contamination. The comparative analysis between the two markers showed that SCoT is more efficient than ISSR. The different performance of the molecular markers may be due to the structure and nature of the marker system and their different targeted genomic regions [75].

The dendrogram based on molecular marker data using UPGMA analysis is an effective tool in numerical computation [80]. The cluster analysis of the SCoT and ISSR markers clearly separated the experimental groups according to the level of crude oil pollution. The two markers showed a similar grouping pattern where the *V. rosea* plants raised in low pollution levels of crude oil were grouped close to control plants, while the opposite is true for plants raised at high levels of contamination, which are grouped further from the control plants and closer to each other. The genetic similarity values obtained from SCoT data showed the highest similarity of 0.91 between the plants raised under low crude pollution oil and the control, while the shortest genetic distance of 0.75 from the control plants was found in plants raised under a high level of crude oil treatment 7%. Likewise, the genetic similarity according to the ISSR marker data ranged from a minimum similarity of 0.72, between plants raised under 7% crude oil treatment and the control, to a maximum similarity of 0.95, between plants raised under 1% crude oil treatment and the control. The heatmap analysis of SCoT and ISSR profiles displayed a clear visual separation among the studied plants raised under different oil treatment levels, specifically between plants raised under the highest level of crude oil treatment (7%) and those under other treatment levels.

There was a treatment-dependent trend observed, as plants raised under high treatment levels of crude oil showed fewer similarities to the control plants; this may be related to the increased number of changes in DNA caused by the increased pollution level. Changes in the number of DNA bands are generally linked to changes in genetic material [81]. As reported by Atienzar et. al. [82], the appearance of new bands may indicate a change in the oligonucleotide priming sites resulting from homologous recombination, mutations, and/or deletions. Similarly, the disappearance of DNA normal bands was prominent in soil polluted with high levels of crude oil. The disappearance of normal bands may be linked to events such as point mutations, DNA damage (e.g., single and double-strand breaks), changed bases, basic sites, oxidized bases, bulky adducts, DNA–protein crosslinks, and/or the complicated chromosomal rearrangements generated by genotoxins [83].

Variations in the GTS of SCoT and ISSR banding patterns may be directly correlated to physiological and metabolic changes [84]. The reduction in GTS% is due to the increase in crude oil pollution and is considered an early molecular indicator of DNA damage [81, 83]. The DNA changes in plants caused by pollution stress were previously confirmed [85, 86]. The treatment-dependent alteration in profiles of molecular markers with decreased GTS was observed in the genomes of some plant species that were exposed to environmental stresses, e.g., *Lemna* species [87], *Ipomoea aquatica* [88], *Pistia stratiotes* [89], *Trifolium repens* [90], *Solanum melongena* [84], *Phaseolus vulgaris* [91]., and *Hordeum vulgare* [92]. Few previous studies have used molecular markers to study the genotoxic effects of crude petroleum oil on plants. This study demonstrated the importance of using molecular markers in the assessment of crude petroleum oil genotoxicity in *V. rosea.*

As demonstrated in this study, *V. rosea* plants can significantly degrade crude oil in soils and may develop response strategies to tolerate the toxic effects caused by crude oil pollution. The plants respond by enhancing or suppressing their functional traits, indicating the ability to survive low concentrations of crude oil pollution by maintaining a balance with the stressed environment.

## Conclusions

The success of phytoremediation of oil-contaminated soils depends on several factors, including environmental conditions, soil characteristics, plant species, and the methodology used in the experiments. The selection of *V. rosea* in this study was based primarily on the plant’s ability to survive the environmental conditions in the region. In addition, the species demonstrated success in the remediation of toxic metal polluted soils as well as the ability to grow in poor soils with a rapid growth rate and a well-developed root system.

The species proved the ability to balance the functional trait variations and to enhance TPH degradation under different levels of crude oil pollution, which are critical to recommend the plant’s use for the phytoremediation of polluted sites. Future research on *V. rosea* should consider the plant–microbe relationships, particularly the rhizosphere microorganisms, to identify and maximize the microbial diversity of oil-degrading microorganisms. The genotoxic effects of crude oil pollution on *V. rosea* still require further investigation. Further studies are required to demonstrate the mechanism of phenolic, flavonoid, and antioxidant compounds in the protection of plants against crude oil pollution stress. Testing different molecular markers and studying the differentially expressed genes will help to identify the genetic polymorphism and stress-resistant genes in response to crude oil pollution.

## Supplementary Information

**Additional file 1: Supplementary Table 1.** (A) Genetic similarity matrix of the SCoT analysis data and (B) of the ISSR analysis for the control and treated *Vinca rosea* plants. C, is the control plants (0% oil); four crude petroleum oil treatments (1%, 3%, 5% and 7%). **Supplementary Table 2.** Change in number of produced bands in SCoT and ISSR profiles of plant samples raised under crude oil treatment levels of 0% (control), 1, 3, 5 and 7%, and genomic template stability (GTS%).

## Data Availability

The datasets used and/or analyzed during the current study are available from the corresponding author on reasonable request.

## Abbreviations

bp: Base pair
DNA: Deoxyribonucleic acid
FRAP: Ferric reducing ability of plasma
GAE: Gallic acid equivalents
GTS: Genomic template stability
ISSR: Inter-simple sequence repeat
μM TE: Micromol trolox equivalents
PCR: Polymerase chain reaction
PIC: Polymorphic information content
RAE: Rutin acid equivalents
SCoT: Start codon targeted
TPH: Total petroleum hydrocarbons
TPTZ: 2,4,6-Tripyridyl-s-triazine
UPGMA: Unweighted pair group method with arithmetic average
