# Supplementary material for: Eco-physiological response and genotoxicity induced by crude petroleum oil in the potential phytoremediator Vinca rosea L
Source: J Genet Eng Biotechnol. 2022 Sep 20;20:135. doi: 10.1186/s43141-022-00412-6 (PMC9489826; doi:10.1186/s43141-022-00412-6)
Supplement: Supplementary file 1 — Additional file 1: Supplementary Table 1. (A) Genetic similarity matrix of the SCoT analysis data and (B) of the ISSR analysis for the control and treated Vinca rosea plants. C, is the control plants (0% oil); four crude petroleum oil treatments (1%, 3%, 5% and 7%). Supplementary Table 2. Change in number of produced bands in SCoT and ISSR profiles of plant samples raised under crude oil treatment levels of 0% (control), 1, 3, 5 and 7%, and genomic template stability (GTS%). [file 43141_2022_412_MOESM1_ESM.docx]

**Supplementary data**

Supplementary Table 1: (A) Genetic similarity matrix of the SCoT analysis data and (B) of the ISSR analysis for the control and treated *Vinca rosea* plants. C, is the control plants (0% oil); four crude petroleum oil treatments (1%, 3%, 5% and 7%).

(A) (B)

|  | **C** | **1%** | **3%** | **5%** | **7%** |  |  | **C** | **1%** | **3%** | **5%** | **7%** |
| --- | --- | --- | --- | --- | --- | --- | --- | --- | --- | --- | --- | --- |
| **C** | **100** |  |  |  |  |  | **C** | **100** |  |  |  |  |
| **1%** | **91** | **100** |  |  |  |  | **1%** | **95** | **100** |  |  |  |
| **3%** | **84** | **87** | **100** |  |  |  | **3%** | **88** | **91** | **100** |  |  |
| **5%** | **78** | **80** | **82** | **100** |  |  | **5%** | **79** | **80** | **87** | **100** |  |
| **7%** | **75** | **77** | **78** | **79** | **100** |  | **7%** | **72** | **75** | **80** | **86** | **100** |

Supplementary Table 2: Change in number of produced bands in SCoT and ISSR profiles of plant samples raised under crude oil treatment levels of 0% (control), 1, 3, 5 and 7%, and genomic template stability (GTS%).

|  | Percentage of oil treatment (oil: soil by mass) | | | | | | | | |  | Percentage of oil treatment (oil: soil by mass) | | | | | | | | |
| --- | --- | --- | --- | --- | --- | --- | --- | --- | --- | --- | --- | --- | --- | --- | --- | --- | --- | --- | --- |
|  | **Control** | **1%** | | **3%** | | **5%** | | **7%** | |  | **Control** | **1%** | | **3%** | | **5%** | | **7%** | |
| SCoT Primer Name | **Total** | **p** | **d** | **p** | **d** | **p** | **d** | **p** | **d** | **ISSR Primer Name** | **Total** | **p** | **d** | **p** | **d** | **p** | **d** | **p** | **d** |
| SCoT-1 | 6 | 0 | 0 | 0 | 0 | 0 | 0 | 0 | 0 | **ISSR-3** | 10 | 0 | 3 | 0 | 3 | 0 | 2 | 0 | 3 |
| SCoT-2 | 4 | 1 | 0 | 1 | 0 | 1 | 1 | 2 | 2 | **ISSR-4** | 5 | 0 | 0 | 0 | 0 | 0 | 2 | 2 | 2 |
| SCoT-3 | 2 | 1 | 0 | 1 | 0 | 1 | 0 | 3 | 0 | **ISSR -5** | 4 | 0 | 0 | 0 | 0 | 0 | 0 | 1 | 2 |
| SCoT-4 | 7 | 0 | 1 | 0 | 1 | 3 | 2 | 2 | 1 | **ISSR -8** | 7 | 0 | 0 | 0 | 0 | 1 | 2 | 2 | 2 |
| SCoT-5 | 11 | 1 | 2 | 2 | 1 | 0 | 3 | 2 | 4 | **ISSR -9** | 5 | 0 | 0 | 0 | 2 | 3 | 2 | 3 | 2 |
| SCoT-6 | 7 | 1 | 3 | 2 | 3 | 4 | 4 | 3 | 3 | **ISSR -13** | 2 | 2 | 0 | 3 | 0 | 5 | 0 | 2 | 0 |
| SCoT-7 | 3 | 0 | 0 | 1 | 0 | 1 | 0 | 3 | 0 | **ISSR -14** | 6 | 0 | 0 | 1 | 0 | 2 | 0 | 1 | 0 |
| SCoT-9 | 11 | 0 | 0 | 0 | 2 | 0 | 2 | 1 | 2 | **ISSR -19** | 10 | 0 | 1 | 0 | 2 | 0 | 2 | 0 | 2 |
| SCoT-10 | 12 | 0 | 1 | 2 | 2 | 0 | 2 | 0 | 2 | **ISSR -20** | 6 | 0 | 0 | 1 | 2 | 1 | 2 | 1 | 2 |
| SCoT-11 | 12 | 1 | 0 | 3 | 3 | 3 | 5 | 2 | 4 | **R -9** | 3 | 0 | 0 | 0 | 0 | 1 | 0 | 4 | 0 |
| total | **75** | **5** | **7** | **12** | **12** | **13** | **19** | **18** | **18** | **total** | **58** | **2** | **4** | **5** | **9** | **13** | **12** | **16** | **15** |
| a |  |  | **12** |  | **24** |  | **32** |  | **36** | **a** |  |  | **6** |  | **14** |  | **25** |  | **31** |
| GTS% | **100** |  | **84** |  | **68** |  | **57** |  | **52** | **GTS%** | **100** |  | **90** |  | **76** |  | **57** |  | **47** |

p= appearance of new bands in treated samples; d= disappearance of bands in treated sample; a is the polymorphism profile (a= p+d); n= total bands.
